# Co-expression of active human cytochrome P450 1A2 and cytochrome P450 reductase on the cell surface of *Escherichia coli*

**DOI:** 10.1186/s12934-016-0427-5

**Published:** 2016-02-02

**Authors:** Paul Quehl, Joel Hollender, Jan Schüürmann, Tatjana Brossette, Ruth Maas, Joachim Jose

**Affiliations:** Institut für Pharmazeutische und Medizinische Chemie, PharmaCampus, Westfälische Wilhelms-Universität Münster, Corrensstraße 48, 48149 Münster, Germany; Autodisplay Biotech GmbH, Merowingerplatz 1a, 40225 Düsseldorf, Germany

**Keywords:** Surface display, Autotransporter, Autodisplay, Cytochrome P450 1A2, Cytochrome P450 reductase, Whole cell biocatalysis

## Abstract

**Background:**

Human cytochrome P450 (CYP) enzymes mediate the first step in the breakdown of most drugs and are strongly involved in drug–drug interactions, drug clearance and activation of prodrugs. Their biocatalytic behavior is a key parameter during drug development which requires preparative synthesis of CYP related drug metabolites. However, recombinant expression of CYP enzymes is a challenging bottleneck for drug metabolite biosynthesis. Therefore, we developed a novel approach by displaying human cytochrome P450 1A2 (CYP1A2) and cytochrome P450 reductase (CPR) on the surface of *Escherichia coli*.

**Results:**

To present human CYP1A2 and CPR on the surface, we employed autodisplay. Both enzymes were displayed on the surface which was demonstrated by protease and antibody accessibility tests. CPR activity was first confirmed with the protein substrate cytochrome c. Cells co-expressing CYP1A2 and CPR were capable of catalyzing the conversion of the known CYP1A2 substrates 7-ethoxyresorufin, phenacetin and the artificial substrate luciferin-MultiCYP, which would not have been possible without interaction of both enzymes. Biocatalytic activity was strongly influenced by the composition of the growth medium. Addition of 5-aminolevulinic acid was necessary to obtain a fully active whole cell biocatalyst and was superior to the addition of heme.

**Conclusion:**

We demonstrated that CYP1A2 and CPR can be co-expressed catalytically active on the cell surface of *E. coli*. It is a promising step towards pharmaceutical applications such as the synthesis of drug metabolites.

**Electronic supplementary material:**

The online version of this article (doi:10.1186/s12934-016-0427-5) contains supplementary material, which is available to authorized users.

## Background

Microsomal cytochrome P450 monooxygenases (CYPs, P450s) are the major enzymes in human drug metabolism as they are involved in the breakdown of almost all marketed drugs [1]. As part of the phase-I-metabolism these heme-containing proteins catalyze a huge variety of oxidation reactions accepting a broad range of endogenous and xenobiotic substrates [2]. They hereby convert lipophilic into more reactive and hydrophilic metabolites as first step for their elimination from the body. Of all 57 human CYPs known, five (CYP3A4, 2C9, 2C19, 2D6 and 1A2) catalyze the vast majority of drug oxidations. For these class II type CYPs the required electrons are supplied from the cytochrome P450 reductase (CPR, also referred to as CYPOR) which transfers them from nicotinamide adenine dinucleotide phosphate (NADPH) through its cofactors flavin adenine dinucleotide (FAD) and flavin mononucleotide (FMN) to the heme of the monooxygenase [3]. CYP enzymes are an essential determinant for the half-life and safety of a drug and its metabolic products. Consequently, they represent a key factor in drug discovery and development. For the complete pharmacological characterization of a drug candidate, its metabolites must be produced on a preparative scale [4, 5]. There is a natural interest in exploiting recombinant CYPs for the synthesis of drug metabolites, in particular since classical chemical synthesis routes are often very challenging and costly [6]. However, as membrane-bound proteins, their heterologous expression and purification is challenging, laborious and often requires membrane preparations to yield active enzymes [7, 8]. For large scale applications, the usage of purified CYPs suffers from low catalytic activities, purification costs and poor enzyme stability.

**Fig. 1:**
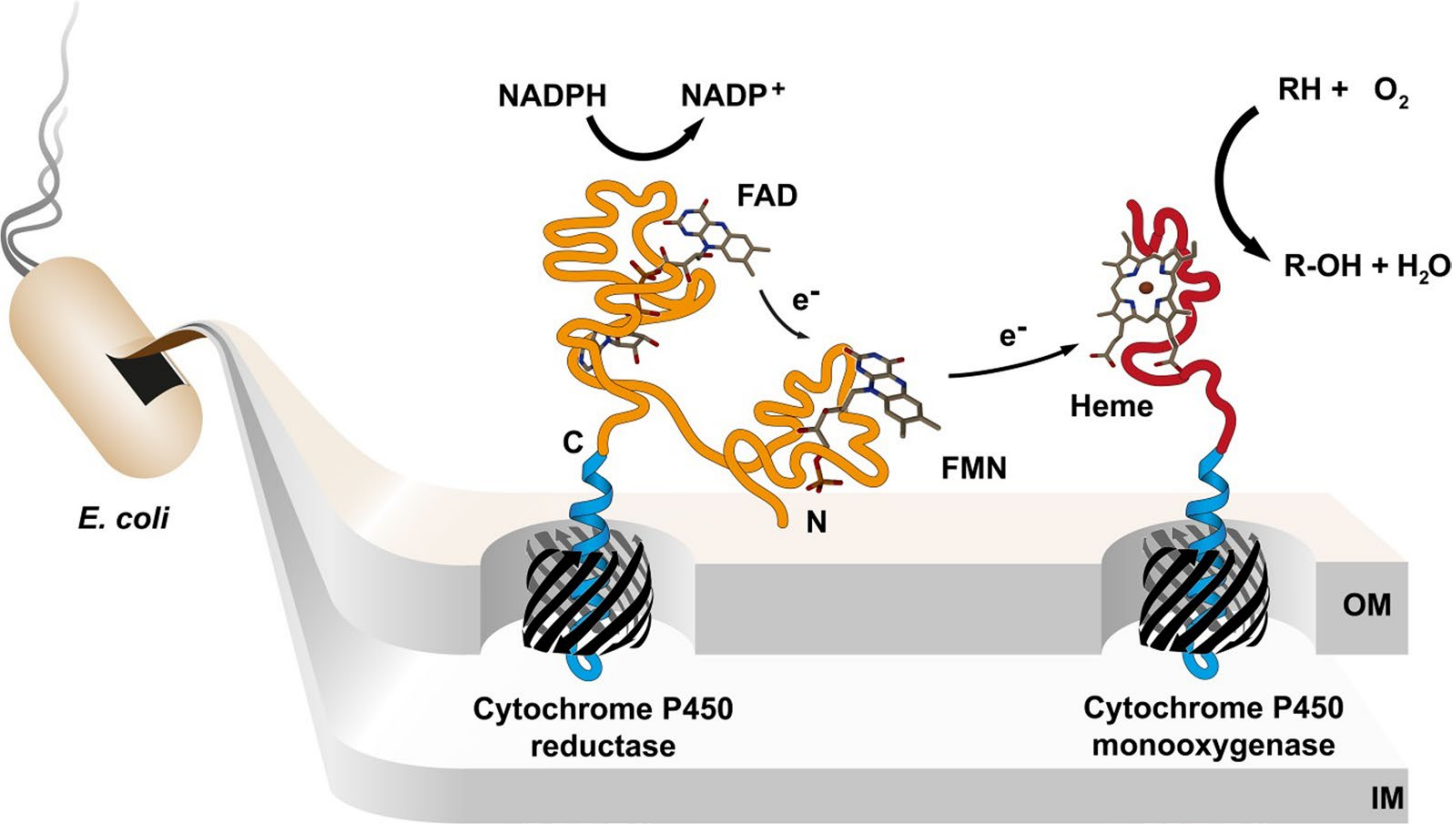
Illustration of the biocatalysis by CYP1A2 and CPR on the cell surface. Two electrons are shuttled by the outer membrane (OM) anchored CPR from NADPH via the cofactors FAD and FMN in single-electron steps to the heme group of surface displayed CYP1A2. The monooxygenase catalyzes the oxidative breakdown of a substrate by inserting one oxygen atom into the chemical compound while reducing the other one to water. *IM* inner membrane

Many of these challenges can be solved by the application of a bacterial whole cell catalyst with surface displayed enzymes. The approach is illustrated in Fig. 1. Anchorage of the enzyme in the outer membrane of *Escherichia coli* provides a membrane environment and circumvents mass transfer limitations due to the membrane barrier. Further major advantages are the cheap and easy cultivation, feasibility of large-scale applications and reusability of the biocatalyst. Additionally, common expression hosts like *E.coli* have no own CYP background. As a biotechnological tool for surface display of recombinant proteins so-called autotransporters have been widely employed [9]. They are derived from natural outer membrane proteins in gram-negative bacteria and their translocation mechanism and structure have been intensively studied [10–14]. The technique has been successfully applied for the display of a variety of enzymes such as nitrilase [15], lipase and foldase [16], protein kinase CK2 [17] as well as other proteins like V_HH_ antibody fragments [18], affibodies [19] and peptides [20]. In this study, we employed the two *E.coli* autotransporters AIDA-I [21] and EhaA [22, 23]. For surface display, the protein of interest (“passenger”) is combined with an N-terminal signal peptide and the C-terminal β-domain (also referred as autotransporter unit) of the autotransporter which consists of the β_1_-(“autochaperone”) domain, α-helix and β-barrel domain [12, 22]. After translation the protein is transported through the Sec-pathway across the inner membrane [14]. The signal peptide is cleaved off and the protein kept in an unfolded confirmation by periplasmic chaperones such as Skp and SurA. The β-barrel is then inserted into the outer membrane with assistance of the Omp85/Bam complex while the passenger is translocated to the extracellular space.

Human CYP1A2 has a molecular weight of 58 kDa including a 29 amino acid long N-terminal transmembrane domain and contains heme b in its catalytic center [24]. Known substrates like phenacetin, paracetamol, coffein and imipramine are mostly planar polyaromatic amides and amines. CYP1A2 catalyzes about 9 % of CYP related drug metabolism [1]. The redox partner protein, the 77 kDa sized human CPR, is composed of a 55 amino acid N-terminal transmembrane domain, a FMN and a FAD/NADPH binding domain which are connected through a flexible hinge region [25, 26]. The CPR undergoes conformational changes between an open and closed form during its redox-cycle, but only the open form can transfer electrons to all microsomal CYPs. CPR is also able to supply electrons to other redox partners such as heme oxygenase and squalene monooxygenase.

Previously, it has been shown that human CYP3A4 can be displayed in an active form on the surface of *E.coli* using the AIDA-I autotransporter [27]. The obtained whole cell biocatalyst was able to convert testosterone into 6β-hydroxytestosterone with externally added CPR and cytochrome b5. Furthermore, soluble bacterial CYP enzymes such as BM3 [28] and CYP106A2 [29] have been expressed on the surface of bacteria and used for biocatalytic studies. Rat CPR alone has been surface displayed on *E.* *coli* using ice-nucleation protein from *Pseudomonas syringae* and was active towards cytochrome c [30]. Displayed on *Bacillus subtilis* spores rat CPR was able to transfer electrons to externally added CYP1A2 which was shown by 7-ethoxyresorufin-O-deethylation [31]. Belonging to the class I P450 system, mitochondrial bovine adrenodoxin has been brought to the surface and was active with its externally added redox partners [32] and in electrochemical analyses [33] when the iron-sulfur protein was reconstituted with supplemented [2Fe–2S] clusters.

In this study, we report on the first successful co-expression of CYP1A2 and CPR on the surface of *E.coli*. We present experimental evidence that this whole cell biocatalyst is functionally active and able to catalyze three typical CYP1A2 oxidation reactions.

## Results

### Vector design

**Fig. 2:**
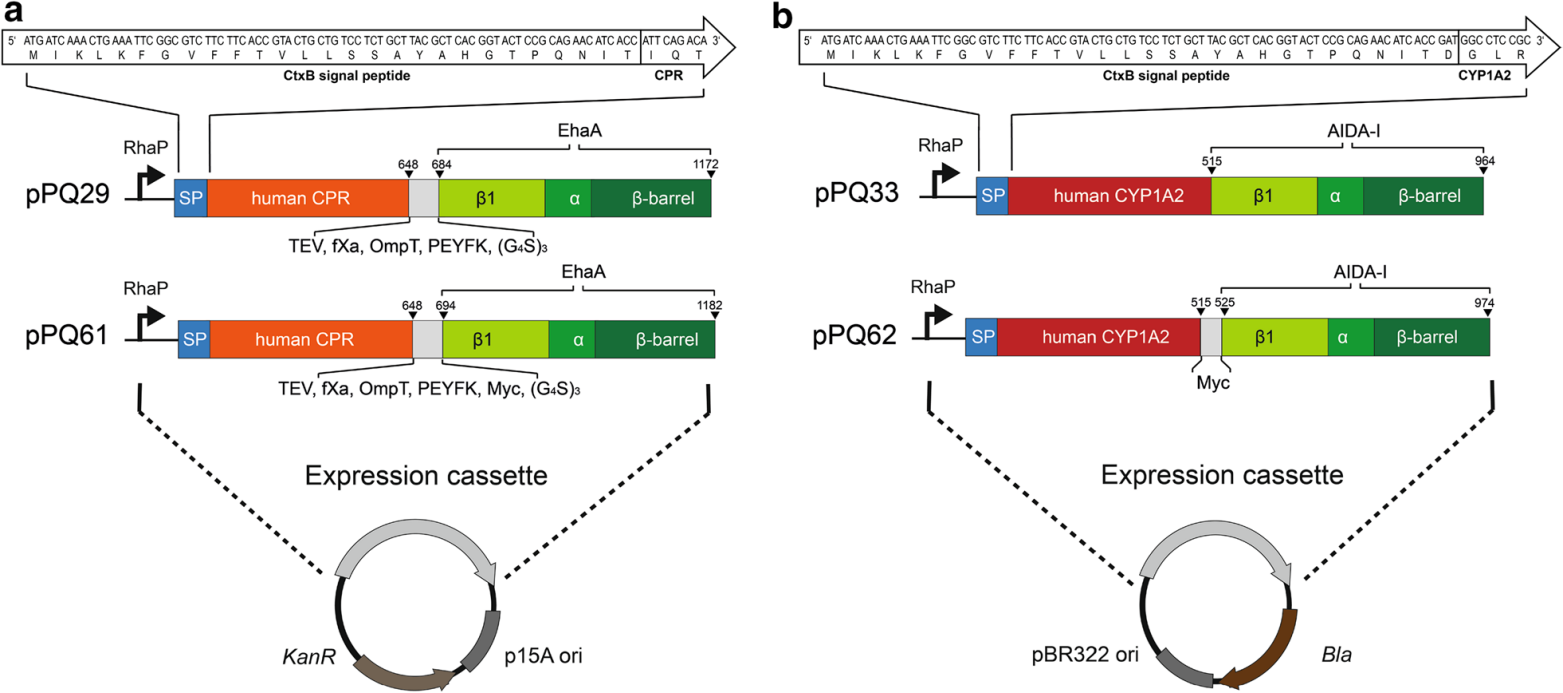
Schematic depictions of the expression vectors for the CPR (**a**) and CYP1A2 (**b**) autotransporter fusion proteins. The expression cassettes consist of the rhamnose inducible promoter (RhaP), the CtxB signal peptide (SP), passenger (CPR: *orange*, CYP1A2: *red*), a connecting region (*grey*) and the C-terminal parts of the autotransporter AIDA-I or EhaA comprising the β_1_-domain (β_1_), α-helix (α) and β-barrel. The connecting region between the CPR and the EhaA is composed of TEV, factor Xa and OmpT protease cleavage sites, a PEYFK epitope and a 15 amino acid long flexible glycine/serine (G_4_S)_3_ part (pPQ29). For flow cytometry analysis the myc epitope (Myc) was inserted in between the passengers and autotransporter unit yielding pPQ61 (CPR) and pPQ62 (CYP1A2). The N-termini of the fusion proteins are expanded as *white arrows* to show the DNA- and amino acid sequences. Plasmids for CYP1A2 autotransporter fusion protein expression (**b**) contained the pBR322 ori and β-lactamase gene as selection marker (*bla*) and plasmids for CPR autotransporter fusion protein expression (**a**) the P15A ori and kanamycin resistance (*KanR*)

Two compatible expression vectors were constructed comprising the human CPR and CYP1A2 coding sequences in combination with the necessary autotransporter elements under control of a rhamnose inducible promoter. The promoter is tightly regulated and expression level is well titratable [34]. The obtained fusion protein consists of an N-terminal CtxB signal peptide for translocation through the Sec pathway, the CPR or CYP1A2 passenger without the transmembrane domains (amino acids 1–56 and 1–29, respectively) and the C-terminal autotransporter unit for outer membrane anchorage (Fig. 2). The CPR was combined with the EhaA and CYP1A2 with the AIDA-I autotransporter unit. For clarity, we address these fusion proteins as CPR and CYP1A2, respectively. Protein expression was always conducted with 1 mmol L^−1^ rhamnose in the OmpT negative *E.coli* strain BL21(DE3).

### Evaluation of surface display by protease accessibility test

**Fig. 3:**
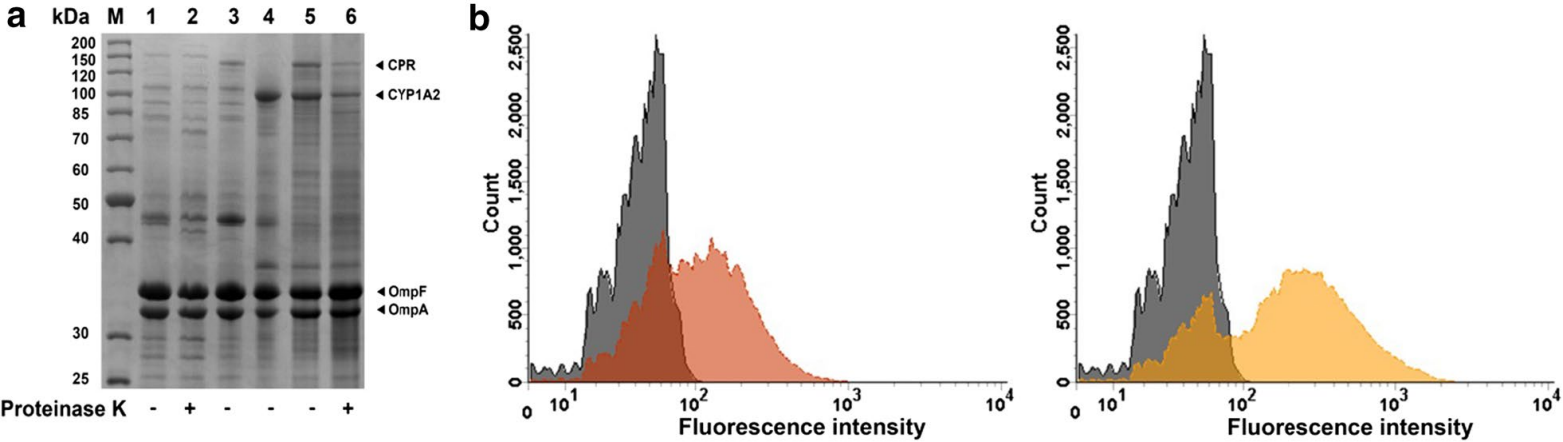
Surface accessibility of CYP1A2 and CPR. **a** SDS-PAGE of outer membrane protein isolations and investigation of protease accessibility. M: Protein marker, apparent molecular weights are indicated on the *left*. *Lane 1–2* sample from *E. coli* BL21(DE3) host cells, *lane 3* sample from cells with induced expression of CPR fusion protein, *lane 4* sample from cells with induced expression of CYP1A2 fusion protein, *lane 5–6* samples from cells with induced co-expression of both fusion proteins. Samples in *lane 2* and *6* were treated with proteinase K prior to outer membrane protein isolation. **b** Flow cytometry analyses of immunolabelled cells. Cell samples were treated with a primary monoclonal anti-myc antibody and a secondary Dylight488 conjugated anti-IgG antibody, washed and then analyzed via flow cytometry. *Grey* *E. coli* BL21(DE3) control cells, *red* cells expressing CYP1A2, *orange* cells expressing CPR

To examine surface expression of the two autotransporter fusion proteins, an outer membrane protein isolation (OMPI) was performed and analyzed by SDS-PAGE. Additionally, the same portion of the cells was treated 1 h at 37 °C with proteinase K prior to the OMPI procedure to investigate the protease accessibility of the passengers. Protease accessibility is a simple experiment to test the surface display of passengers as the protease is regarded to be too large to pass the outer membrane and digests only proteins exposed on the cell surface. The results are shown in Fig. 3. Samples of cells expressing either the CYP1A2 or CPR fusion protein contained a distinct protein band around the expected apparent molecular weight of 103 and 127 kDa, respectively. No comparable band was found in the control sample. In the OMPI sample of cells co-expressing the CPR and CYP1A2 fusion proteins both protein bands were also detectable. CYP1A2 is moderately higher expressed likely due to its smaller size and the higher plasmid copy number of the pBR322 origin of replication (ORI) in comparison to the P15A ORI (15–20 to ~10 copies) used for CPR expression. This would suggest a CYP1A2:CPR ratio above 1, if all enzymes were active. Both bands were substantially reduced when cells were treated with proteinase K prior to OMPI. This demonstrates a clear protease accessibility of the passengers which is a strong indication for surface exposure of the passengers. The native outer membrane protein OmpA functions as an internal control for excessive protease treatment. If proteinase K had entered the periplasm, the periplasmic domain of OmpA would have been cleaved off. This can be detected by disappearance of the OmpA protein band, which, however, was unchanged in our experiments. In conclusion, the degradation of autotransporter fusion proteins and the intactness of OmpA suggest a successful surface display of CYP1A2 and CPR.

### Evaluation of surface display by flow cytometry and immunofluorescence microscopy

Flow cytometry is a common technique to investigate surface display of a protein of interest. An antibody does not penetrate the outer membrane due to its size and only binds to proteins exposed on the cell surface. Only for this assay the myc-tag had been genetically introduced in between the passenger and the autotransporter unit (Fig. 2). Cells were treated with a myc-tag specific primary antibody and a Dylight488 conjugated secondary antibody and fluorescence intensities of the cells analyzed subsequently via flow cytometry. Fluorescence intensities of cells expressing the CYP1A2 (pPQ62) or CPR (pPQ61) (Fig. 3b) were significantly higher in comparison to the fluorescence intensity of host cells without plasmid indicating the surface exposure of both proteins. To confirm these results immunofluorescence microscopy analysis of these cells were performed. For this purpose, mere host cells, cells expressing CPR with myc-tag (pPQ61, Fig. 2) and cells expressing CYP1A2 with myc-tag (pPQ62, Fig. 2) were treated with a mouse anti-myc antibody and a secondary Dyligh488 conjugated anti-IgG antibody and subsequently subjected to confocal laser scanning microscopy. Whereas mere host cells did not exhibit immunofluorescence after this treatment, CPR and CYP1A2 expressing cells clearly showed such fluorescence (Additional file 1: Figure S1). When the fluorescence exhibiting cells were treated with proteinase K before the antibodies were added, immunofluorescence almost completely disappeared (S1). This strongly supported the results obtained by flow cytometry and indicated that CPR and CYP1A2 were indeed surface-expressed. Introduction of the myc-tag did not change the level of protein expression as assessed by OMPI. Introduction of the myc-tag did also not influence interaction of CPR and CYP1A2. For simplification, however, all following experiments were performed with the passengers without myc-tag.

### Cytochrome c activity

**Fig. 4:**
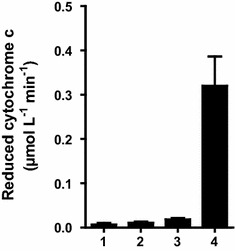
Cytochrome c activity. Cytochrome c reduction by whole cells (OD_578nm_ 0.25) was measured at 550 nm. Reaction was started by addition of 100 µmol L^−1^ NADPH. (1) Assay without cells, (2) *E.* *coli* BL21(DE3) cells, (3) cells expressing the CYP1A2, (4) cells expressing CPR

First, we investigated the activity of single expressed CPR. The reduction of the 11.8 kDa substrate protein cytochrome c is a standard assay to investigate the enzymatic activity of a CPR [35]. Following NADPH oxidation the CPR transfers one electron to cytochrome c which can be detected by an absorbance increase at 550 nm. The assay was conducted with washed cells (OD_578nm_ 0.25), 100 µmol L^−1^ NADPH and 50 µmol L^−1^ cytochrome c. The results are shown in Fig. 4. Cells expressing CPR exhibit an activity of about 0.32 µmol L^−1^ min^−1^ whereas neither the host cells without plasmid nor cells expressing CYP1A2 on the surface without CPR showed any significant activity in comparison to the NADPH control without cells. This demonstrates that surface displayed CPR binds its cofactors FAD and FMN and hence is capable to shuttle electrons from NADPH to cytochrome c. Moreover, cytochrome c is not membrane permeable. Thus, these results support that the CPR was displayed on the cell surface.

### 7-Ethoxyresorufin-O-deethylation activity

**Fig. 5:**
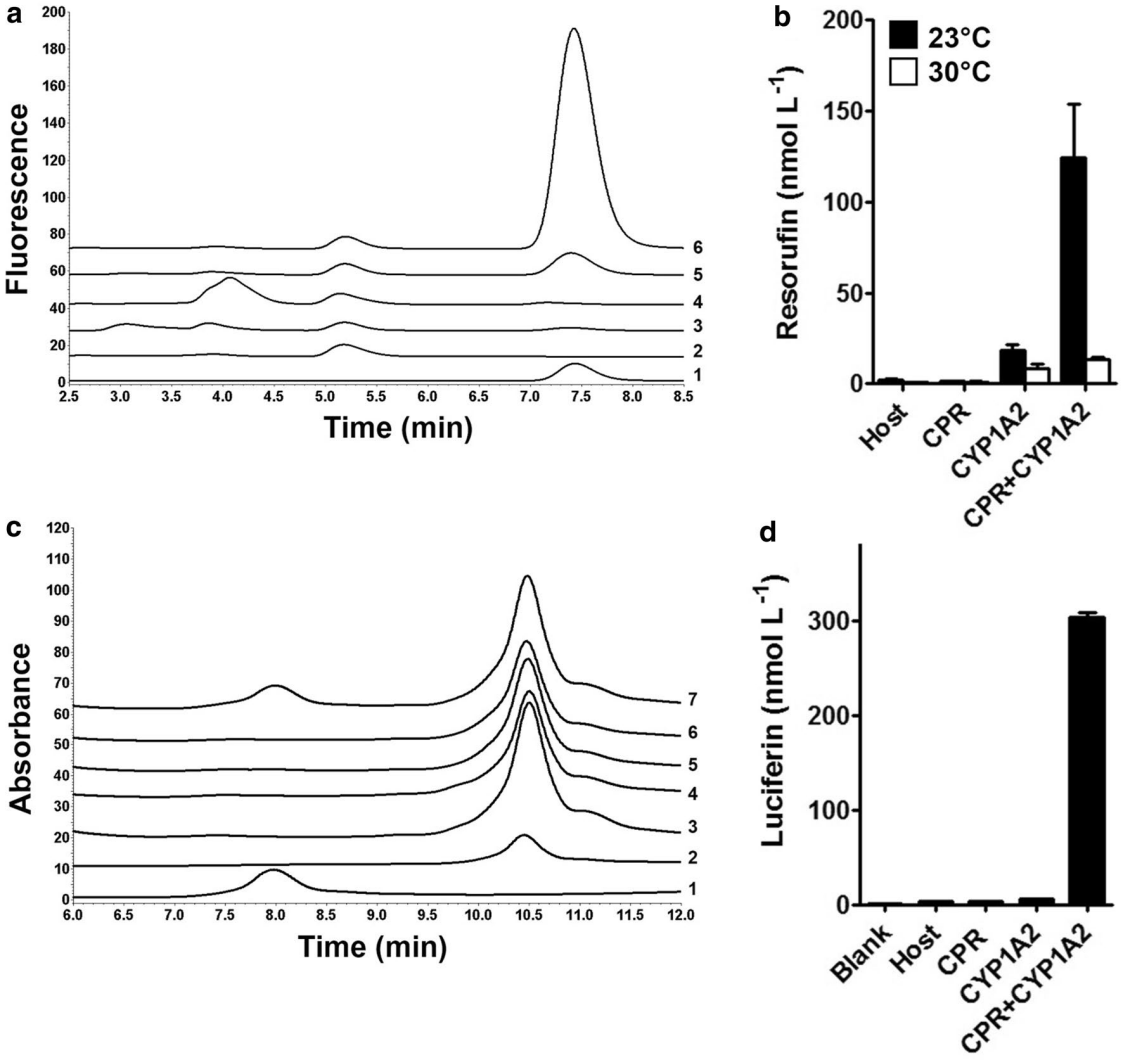
Conversion of known CYP1A2 substrates. **a** HPLC chromatogram of samples from the 7-ethoxyresorufin-O-deethylation assay. Samples were diluted 1:1 with methanol prior to HPLC analysis. (1) Resorufin standard (10 nmol L^−1^), (2) co-expression sample without substrate in the assay, (3) host cells, (4) cells displaying CPR, (5) cells displaying CYP1A2, (6) cells expressing CYP1A2 and CPR. **b** Resorufin formation in the whole assay determined by HPLC following expression at 23 or 30 °C. **c** HPLC chromatogram of samples of the phenacetin-O-deethylation assay. (1) Paracetamol standard (10 µmol L^−1^) with 8.0 min retention time, (2) 3-acetamidophenol internal standard (10 µmol L^−1^) with 10.5 min retention time, (3) control sample with co-expression cells without substrate, (4) host cells, (5) cells displaying CPR, (6) cells displaying CYP1A2, (7) cells displaying CYP1A2 and CPR. **d** MultiCYP activity measured by luciferin formation. Blank: Control sample without cells

CYP1A2 catalyzes the conversion of 7-ethoxyresorufin to the highly fluorescent product resorufin, if electrons are supplied by the redox partner protein CPR. We first determined resorufin by HPLC analysis to ensure detection of the product in the assay at low concentrations. Cells were cultivated as described in a sodium/potassium phosphate buffered LB-medium for 19.5 h at 23 °C. Assays were conducted with washed cells (OD_578nm_ 40), 1 µmol L^−1^ 7-ethoxyresorufin and a glucose-6-phosphate dehydrogenase NADPH regeneration system in 0.1 mol L^−1^ potassium phosphate (pH 7.4) for 40 h. An exemplary chromatogram is depicted in Fig. 5a with resorufin appearing at 7.4 min. From the areas under the curve resorufin concentrations were calculated and are shown in Fig. 5b. For host cells without surface displayed enzymes and cells with surface displayed CPR alone resorufin concentration was below 2 nmol L^−1^ at all conditions examined. For cells with co-expression of CYP1A2 and CPR, significant amounts of resorufin were detectable, with 124 nmol L^−1^ resorufin in case of an expression at 23 °C. This clearly indicates that surface displayed CYP1A2 is catalytic active when it is co-expressed with surface displayed CPR. An almost tenfold decrease in activity was observed at an expression temperature of 30 °C (14 nmol L^−1^ resorufin), indicating that enzymatic activity depended strongly on the temperature during expression. A lower expression temperature was favorable which has been reported for CYP enzymes expressed in bacteria in several studies before and appeared to contribute to better folding [7]. The protein expression level of CYP1A2 was obviously increased at 30 °C in comparison to 23 °C as shown by the OMPI samples (Additional file 2: Figure S2 A), demonstrating that the lower activity as observed is not caused by a lower expression rate, but rather due to an unfavorable protein folding. In contrast, expression of CPR was unchanged in comparison to expression at 23 °C. Both passengers were completely accessible by proteinase K, indicating that these proteins were displayed at the cell surface. To our surprise, cells expressing CYP1A2 alone were also active but on a low level from 9 nmol L^−1^ (when expressed at 30 °C) to 19 nmol L^−1^ (at 23 °C).

### Effect of growth medium ingredients on activity

**Fig. 6:**
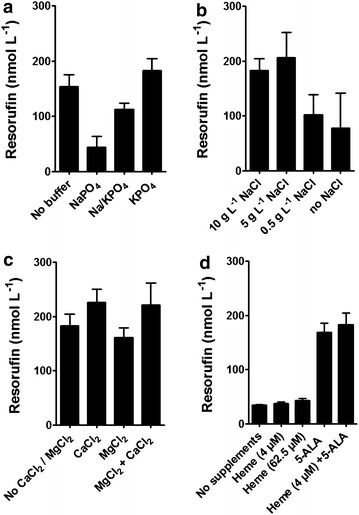
Effect of growth medium components on 7-ethoxyresorufin-O-deethylation. **a** Effect of 50 mmol L^−1^ phosphate buffer in LB medium with 500 µmol L^−1^ 5-ALA and 4 µmol L^−1^ heme. NaPO_4_: sodium phosphate buffer, Na/KPO_4_: mixed sodium/potassium phosphate buffer (37 mmol L^−1^ Na^+^/12 mmol L^−1^ K^+^), KPO_4_: potassium phosphate buffer. **b** Variation of NaCl content in 50 mmol L^−1^ potassium phosphate buffered LB supplemented with 500 µmol L^−1^ 5-ALA and 4 µmol L^−1^ heme. **c** Effect of CaCl_2_ and MgCl_2_ (each 1 mmol L^−1^) in potassium phosphate buffered LB medium supplemented with 500 µmol L^−1^ 5-ALA and 4 µmol L^−1^ heme. **d** Effect of heme and 5-ALA supplementation. Protein expression was conducted in potassium phosphate buffered LB medium with either 4 µmol L^−1^ heme, 62.5 µmol L^−1^ heme, 500 µmol L^−1^ 5-ALA or 500 µmol L^−1^ 5-ALA and 4 µmol L^−1^ heme

It has been reported for purified CYP enzymes that the concentrations of mono- and divalent cationic ions and the ionic strength can induce conformational changes in the protein structure, affect membrane insertion and influence the interaction between partner proteins with a significant impact on activity [36–41]. In case of our novel biocatalyst, folding takes place outside the cell on the outer membrane and could be influenced by the surrounding medium. In consequence, the salt composition of the growth medium would have an effect on activity. To investigate this effect, potassium and sodium phosphate buffered LB-medium as well as several buffered LB medium variants with different NaCl concentrations were tested and—as a read out—the resorufin production of cells grown in these different media was determined. Standard LB-medium is composed of 10 g L^−1^ NaCl, 5 g L^−1^ yeast extract and 10 g L^−1^ peptone. We determined the reaction product resorufin in the corresponding sample supernatants using a microplate reader and set the results into relation to the components added to LB (Fig. 6). As shown in Fig. 6a, there was a severe effect of the buffer used on resorufin production. Cells cultivated in LB medium buffered with 50 mmol L^−1^ sodium phosphate buffer were fourfold less active (44 nmol L^−1^ resorufin) than the equivalent cells grown with potassium phosphate buffer (182 nmol L^−1^ resorufin), and still 2.5 fold less active than the equivalent with a mixed sodium/potassium phosphate buffer (112 nmol L^−1^ resorufin). In this case, increasing the Na^+^ concentration appeared to have a negative effect. However, as shown in Fig. 6b, varying the NaCl content of potassium phosphate buffered LB medium resulted in the highest product titer with 5 g L^−1^ NaCl. Less than 5 g L^−1^ NaCl led to a significant reduction in resorufin production. These results suggest that the NaCl concentration is a critical parameter for defining an optimal growth medium. In our experiments potassium buffered LB medium containing 5 g L^−1^ NaCl turned out to be the growth medium yielding the highest enzymatic activity of the whole cell biocatalyst towards resorufin.

The effects of 1 mmol L^−1^ CaCl_2_ and/or MgCl_2_ as medium additives were also studied. The results are shown in Fig. 6c. Addition of MgCl_2_ to LB medium had no effect on product titer and thus CYP1A2 activity. In contrast, supplementation of the growth medium with CaCl_2_ led to an increase in resorufin production by approximately 30 %. None of the tested conditions altered the level of protein expression as analyzed by OMPI (Additional file 2: Figure S2 B). In summary, our results indicate that the activity of surface displayed CYP enzyme is strongly influenced by the concentrations of Na^+^, and Ca^2+^, as well as the buffer used for the growth medium. One possible reason could be that these alterations influence protein folding or membrane insertion as it has been reported before for purified CYPs [41, 56]. Similar observations have not been reported yet for any other recombinant surface displayed enzyme.

Secondly, we were interested how heme b needs to be provided for a fully functional CYP1A2. The outer membrane is considered to be an impermeable barrier for heme. Although LB medium contains about 5 µmol L^−1^ heme [42], *E.coli* BL21(DE3) is also incapable of actively taking it up due to a lack of heme uptake transporters in the outer membrane [43–45]. It was previously found that surface displayed CYP3A4 and CYP106A2 were active without external addition of heme [27, 29]. Hence, we investigated if supplementation of the precursor 5-aminolevulinic acid (5-ALA) affects the enzymatic activity of the whole cell biocatalyst and if it is a limiting factor. Heme is synthesized in the cytoplasm from eight 5-ALA molecules and can be translocated to the periplasm (e.g. for periplasmic cytochrome c maturation [46]) and to the cell exterior through the TolC channel [47, 48]. For this experiment, potassium phosphate buffered LB medium (as control) or potassium phosphate buffered LB either supplemented with 62.5 µmol L^−1^ hemin chloride, or with 500 µmol L^−1^ 5-ALA or with the standard supplementation of 500 µmol L^−1^ 5-ALA and 4 µmol L^−1^ hemin chloride was used. Hemin chloride is the corresponding salt of heme b. As shown in Fig. 6d, resorufin was detectable using co-expression cells grown in LB medium without supplementation. Addition of hemin led to a 25 % increase in activity in comparison to non-supplemented LB medium. In contrast, addition of the equivalent amount of 5-ALA increased the activity by 400 % to 169 nmol L^−1^ resorufin. Thus, CYP1A2 activity was effectively enhanced through the route of endogenous biosynthesis of heme but hardly through addition of external heme. Although there is little knowledge about the cellular mechanism of cofactor insertion into surface displayed enzymes, the results of this approach clearly indicated that 5-ALA is a key medium component and should be considered in case of future bioprocess optimization.

### MultiCYP activity

To test a second common substrate, we applied the so-called MultiCYP assay (Promega). The measurement of activity relies on the O-demethylation of the MultiCYP substrate to a proluminescent d-luciferin ester, which can then be detected by luminescence after the addition of an esterase and a luciferase containing detection reagent (LDR). Washed cells expressing surface displayed CPR, CYP1A2 or both were suspended in the MultiCYP substrate. After 24 h of incubation the formation of D-luciferinester was determined (Fig. 5d). For this purpose the resulting luminescence was compared to the luminescence values of a d-luciferin calibration curve (not shown). Cells with surface displayed CYP1A2 and CPR produced a significant higher luminescence signal in comparison to cells with only CYP1A2 or CPR, and also in comparison to cells with no plasmid, or control samples without any cells. These results underline that CYP1A2 and CPR were functionally co-expressed and it indicates that the MultiCYP assay represents a viable method for measuring whole cell activity with surface displayed CYPs.

### Phenacetin-O-deethylation activity

The oxidation of phenacetin to the analgesic drug paracetamol is a common first pass reaction and can be used to monitor CYP1A2 activity. To examine the reaction, a whole cell assay was performed with 100 µmol L^−1^ phenacetin as substrate. After the reaction was stopped, samples were spiked with 10 µmol L^−1^ 3-acetamidophenol as internal standard, subsequently extracted with diethylether and analyzed by HPLC. The chromatogram is shown in Fig. 5c. Only the sample of cells co-expressing CYP1A2 and CPR showed a peak at the expected retention time for paracetamol (8 min). Paracetamol concentration was calculated from the calibration curve to be 1.2 µmol L^−1^. No peak was observed at this retention time in any control. It could be a hint that the substrate spectrum of the whole cell biocatalyst was not altered in comparison to soluble CYP1A2, as at least three known substrates of the free enzyme were converted by the whole cell biocatalyst co-displaying CYP1A2 and CPR.

### Time dependent 7-ethoxyresorufin-O-deethylation by the whole cell biocatalyst

**Fig. 7:**
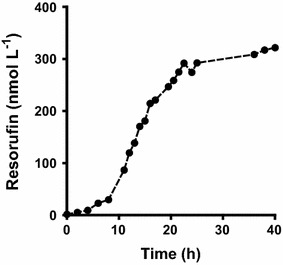
Time dependent characterization of 7-ethoxyresorufin-O-deethylation activity. The whole cell assay was performed with 1 µmol L^−1^ 7-ethoxyresorufin and NADPH regeneration in a 100 mL shake flash with 8 mL starting volume. Concentration of the product resorufin was determined in the sample supernatants. Data points are the mean of at least two independent biological replica and the fluctuation for each data point was for all cases <15 %

Finally, the formation of resorufin was analyzed over a time course of 40 h (Fig. 7). For this purpose the assay was conducted in 100 mL shake flasks containing 8 mL starting volume with 1 µmol L^−1^ of substrate under vigorous shaking at 200 rpm at 37 °C. Cells had been cultivated in potassium phosphate buffered LB-medium supplemented with heme, 5-ALA and CaCl_2_. At the different time points, resorufin concentration was determined in the supernatant of sample aliquots after depleting the cells. The whole cell biocatalyst turned out to be active for about 20 h. The final product concentration was 320 nmol L^−1^ and as such moderately higher than the product concentration obtained in the 15 mL tube-based assay as shown in Fig. 6b. This could have been due to a better oxygen supply in the 100 mL shake flasks. When cells were harvested after 40 h, washed, adjusted to the correct OD and reused in a completely fresh assay, they did not show any enzymatic activity at all.

## Discussion

In our proof of concept study, we report the first successful co-expression of active human CYP1A2 and CPR on the surface of *E. coli* using autodisplay. We found that the cells co-expressing CYP1A2 and CPR on the surface were able to catalyze the conversion of three structurally different substrates (7-ethoxyresorufin, MultiCYP and phenacetin). However, the observed activities were quite low. In our case, the biocatalytic activity would be equivalent to an average of 175 active CYP1A2 enzymes per cell for the phenacetin-O-deethylation. This calculation is based on several assumptions. First it was assumed the measured end-point concentration at 20 h corresponded to the initial maximal velocity of the reaction, and second, that the turnover number was unchanged during this course. For purified human wild type CYP1A2, a k_cat_ value of 1 min^−1^ with a K_M_ of 10 µmol L^−1^ for the phenacetin-O-deethylation has been reported [49]. The number of cells in our assay was calculated to be 3.44 × 10^9^ cells mL^−1^ based on the previously published number of 0.86 × 10^8^ cells per OD and mL [17]. Hence, the turnover per cell would equal 2.9 × 10^−13^ nmol min^−1^ for a 20 h reaction time. This is only a rough estimation, but it can help to evaluate the efficiency of the whole cell biocatalyst. In an analogous calculation, the 7-ethoxyresorufin-O-deethylation activity would equal 22 active CYP1A2 enzymes per cell. In comparison to other whole cell biocatalyst systems, reported values for a whole cell assay with intracellularly expressed CYP1A2 and CPR were 88 mg L^−1^ paracetamol with 100 g L^−1^ cell dry weight within 40 h at 28 °C which is roughly about 70 fold better than our system [50]. In contrast, the observed cytochrome c activity for single expressed CPR would equal about 3000–4700 enzymes per cell. For the cytochrome c reduction by the CPR, a k_cat_ value of 3000 min^−1^ had been reported for the wild type enzyme and a k_cat_ value of 1900 min^−1^ for the anchorless CPR [51]. This number lies within the usual range from 10^3^ to 10^5^ enzymes per cell reported for autodisplay [52, 53]. Nevertheless, cytochrome c activity of CPR can obviously not serve as a predictor for the catalytic activity of CPR with CYP1A2, because of the different binding sites for both proteins with CPR [25, 26, 37]. When comparing Fig. 3 with Fig. 7, there is a clear discrepancy between the amount of protein expressed and CYP1A2 enzymatic activity. This could have been due either to a reduced functionality of surface displayed CYP1A2 molecules or to a restricted interaction between CPR and CYP1A2 on the cell surface. But indeed both would represent major bottlenecks for the application of cells with surface displayed CPR and CYP1A2. An indication for an altered CYP–CPR interaction would be the finding, that truncated and hence soluble CPR is almost unable to interact with CYPs, but is almost as active towards cytochrome c as the complete enzyme [26, 51]. Several reasons need to be taken into account for the presumably reduced activity of CYP1A2, independent of CPR binding. First, the C-terminus is attached to the autotransporter domains which could influence folding. Second, deletion of the N-terminal transmembrane domain, which is necessary in the autodisplay approach could influence folding. And finally, CYP1A2 could misfold to some extent during transport leading to a reduced number of enzymatically active molecules on the cell surface.

Modifying or omitting the N-terminal transmembrane domain is a common step in increasing the expression and solubility of CYPs [8]. It has been proposed that the transmembrane domain does not take part in catalysis, but has the important role to bring CYP and CPR close to the phospholipid interface [54, 55]. The membrane surrounding modulates enzymatic activity as CYP enzymes are partially immersed in the membrane [56, 57]. Several studies about CYP1A2 investigated the effect of a truncated or modified transmembrane domain and did not observe a loss of function, but in some cases different catalytic rates [49, 55, 58]. In contrast, it has been reported for CPR that the soluble truncated form is not able to supply electrons to the monooxygenase [59]. Here, we omitted the N-terminal transmembrane domain of both enzymes to achieve high expression levels. We did not observe a loss of function of the enzymes which supports the hypothesis that the transmembrane domain is not essential for the catalytic activity. Furthermore, CYP1A2 and CPR form a catalytically active unit, although membrane anchorage has been altered from the N- to the C-terminus in both enzymes because of the C-terminal membrane anchoring β-barrel used in autodisplay. Hence, the unusual C-terminal fusion is feasible for both proteins, but our results indicate that this alteration could have modified catalytic properties of the enzymes.

The catalytic activity of the whole cell biocatalyst implies that CPR is able to interact with CYP1A2 and to supply electrons through a transient protein–protein binding. This supports the theory that autotransporter facilitated anchorage allows lateral movement of the passengers on the outer membrane and quaternary complex assembly of protein partners. For other surface displayed proteins, it has been reported that multimerization is feasible [15, 17]. Conclusively, we demonstrated that bacterial surface display facilitates also transient interaction between protein partners.

We identified several factors for expression which influences activity of the whole cell biocatalyst and could be promising parameters for bioprocess optimization. The whole cell biocatalyst was tenfold more active when grown at 23 °C instead of 30 °C. It has been often observed for CYP enzymes that the choice of expression temperature was highly important [7]. For surface displayed proteins, lower temperatures can also be favorable [60]. Furthermore, we found that Na^+^ and Ca^2+^ concentrations in the growth medium affected the product titer of washed cells without affecting the protein expression level. Addition of 1 mmol L^−1^ of CaCl_2_—but not MgCl_2_—was beneficial as the product titer of resorufin increased by roughly 30 %. Na^+^ concentration in the growth medium strongly affected CYP1A2 activity. As intracellular Na^+^ and Ca^2+^ concentrations are actively maintained in living *E.coli* cells, it appears likely that processes in the periplasm or on the outer membrane are affected (e.g. protein folding or membrane insertion of the CYP enzymes) as they are directly accessible to changes in the medium composition. Nevertheless, only few studies have investigated the influence of medium parameters for surface displayed proteins on *E.coli* [60], and hence much more experiments are necessary to get a deeper insight in the influence of growth factors on the efficiency of surface display. But it needs to be taken into consideration that an important step towards the setting up of a reliable bioprocess with surface displayed enzymes will be to determine the optimal medium for cell cultivation.

To investigate if the supply of cofactors is a limiting factor for the activity of the whole cell biocatalyst, we focused exemplarily on heme as it is derived from only one precursor which can be easily added to the medium. *E. coli* BL21(DE3) is able to completely synthesize heme from glutamate via 5-ALA in the cytosol and to transport it to the periplasm [46]. The precursor 5-ALA is a common additive for expression of CYP enzymes [7]. Cells co-expressing CYP1A2 and CPR showed resorufin activity already in unmodified LB medium which was also found for surface displayed CYP3A4. However, medium supplementation with 5-ALA had a strong positive effect on CYP activity and our data suggests that the intracellular biosynthesis of the prosthetic group is a highly limiting factor. In our study, the route of endogenous heme was by far more effective than external addition of heme. 500 µmol L^−1^ 5-ALA increased CYP activity by 400 %. In contrast, the equivalent amount of exogenous heme had a positive effect of merely 25 %. Secondly, it indicates that *E.coli* is metabolically limited during expression in terms of producing sufficient heme from glutamate. Therefore, this could be a promising starting point for strain engineering.

It remains an open question at what stage during translocation the surface displayed CYP1A2 and CPR incorporate their respective cofactors. One hypothesis is that they are incorporated after translocation onto the outer membrane and taken from the extracellular medium. A second is that they are bound by an at least partially folded passenger in the periplasm and then translocated. It is controversially discussed whether a partially folded passenger can be translocated across the outer membrane. Two mechanisms for translocation—the hairpin model and the Omp85 model—have been proposed whereas only the latter would be compatible with partial protein folding [10]. We observed that heme derived from cells is way more effective for increasing CYP activity than externally added heme. This could indicate that heme is incorporated into CYPs in the periplasm and not scavenged from the medium. However, it has been suggested that the TolC efflux pump provides intracellularly produced heme by exporting it to the extracellular medium from where it can be incorporated into CYPs. The outer membrane protein TolC forms a channel with efflux pumps in the inner membrane [61]. Thereby, porphyrins can be exported from the cytosol to the extracellular space which has been observed when natural heme homeostasis was disrupted [47, 48]. Nevertheless, this hypothesis does not explain why only heme provided by the TolC channel but not by medium supplementation would be effectively scavenged from the extracellular medium. In case of the CPR, there is little knowledge about efflux mechanisms for flavins to the cell exterior and periplasm. Although present in LB-medium, *E. coli* cannot take up flavins [62] and synthesizes them instead in the cytosol from GTP and ribulose-5-phosphate [63]. There are no reports on flavin transporters to the periplasm as periplasmic flavoenzymes are uncommon. However, the YeeO multidrug efflux transporter has been reported to export FAD and FMN to the medium [64]. Thus, it is imaginable that cofactor incorporation takes place on the cell surface after translocation. Still, the mechanism of incorporation needs further investigation.

Nevertheless, for preparative drug metabolite synthesis purposes several issues need to be addressed. First, the optimal CYP/CPR ratio should be determined and the influence of the N-terminal transmembrane domain further tested to improve the biocatalytic activity. Moreover, it could be investigated if the usage of inversed autotransporter (type Ve) is beneficial in terms of the catalytic properties of surface displayed enzymes. In this case the passenger is linked by its N-terminus to the anchorage protein which corresponds to the orientation of the natural transmembrane domain in the endoplasmic reticulum. Secondly, we hypothesize that the activity of the whole cell biocatalyst can be reasonably increased by a thorough bioprocess optimization in terms of growth medium, oxygen supply and NADPH regeneration. Thirdly, the co-expression of cytochrome b5 could be beneficial for the activity of at least some monooxygenases [65]. Fourthly, CYP1A2 is involved in the metabolism of only a small fraction of all drugs. Therefore, an important step will be to establish co-expression of the CPR with all other major drug-metabolizing CYPs.

## Conclusion

For the first time, human CYP1A2 has been successfully co-displayed with its reductase CPR on the surface of *E. coli* using the autotransporter system. It demonstrates that bacterial surface display is a viable tool for obtaining active CYP enzymes. This is an important step toward metabolite screening and preparative drug metabolite synthesis in a whole cell approach regardless of their membrane permeability. It also offers the chance to apply flow cytometry for human CYPs for applications such as enzyme library screening or to develop CYP based biosensors.

## Methods

### Vector construction

The codon-optimized open reading frames (ORF) of CYP1A2 [Uniprot:P05177-2] and CPR [Uniprot:P16435] were obtained by artificial gene synthesis (GeneArt, Regensburg, Germany). For cloning the ligase-free In-Fusion technique was used [66]. *Escherichia coli* strain DH5α was used for all subcloning work. To construct the plasmid encoding the CYP1A2-autotransporter fusion protein the sequence of the autotransporter unit of AIDA-I including the sequence for N-terminal cholera toxin B signal peptide (CtxB) was amplified via PCR from pSC001 [27] and cloned behind the rhamnose-inducible promoter into the pJOE2775 vector [34, 67]. The coding sequence of CYP1A2 without the transmembrane domain (amino acid residues 1–29) was then fused between the CtxB and the AIDA-I autotransporter unit DNA sequence yielding pPQ33. An overview about the fusion protein constructs is given in Fig. 2. For the CPR autotransporter fusion protein expression vector, the origin of replication (ORI) and ampicillin selection marker of pPQ33 were exchanged by the P15A ORI from pKE19 and a kanamycin selection marker from the broad-host range vector pBBR1-MCS2 [68]. The CPR ORF without transmembrane domain (amino acid residues 1–56) was inserted between the CtxB and the codon-optimized EhaA autotransporter unit (amino acids 839–1327 of the native protein) DNA sequence. The latter was taken from pMATE-MT004 [23] and modified to contain a TEV, fXa and OmpT cleavage site, the PEYFK epitope and an additional flexible (G_4_S)_3_ hinge region. The plasmid was termed pPQ29. By inserting the sequence for the anti-myc epitope tag between the passenger and the autotransporter unit into pPQ29 and pPQ33 the plasmids pPQ61 and pPQ62 were obtained. The expression cassettes of all plasmids were checked by sequencing. *E. coli* BL21(DE3) was used for expression experiments.

### Growth medium and culture conditions

Cultivation were performed in lysogeny broth (LB) medium containing 10 g L^−1^ peptone, 5 g L^−1^ yeast extract and 10 g L^−1^ NaCl. Solid media were prepared by addition of 1.5 % (w/v) agar. Kanamycin and carbenicillin were supplemented to ensure plasmid stability to a final concentration of 62.5 and 125 µg mL^−1^, respectively. Precultures were performed in unmodified LB medium at 37 °C overnight. For expression studies LB medium was modified. Depending on the experiment LB was buffered with either a sodium phosphate buffer, a 37:12 mixed sodium/potassium phosphate buffer or a potassium phosphate buffer, each set to pH 7 with a final concentration of 50 mmol L^−1^ phosphate. Protein expression was induced with 1 mmol L^−1^l-rhamnose. Using an autoinduction approach, 0.5 g L^−1^ glucose as catabolite repressor was added delaying expression until approximately OD_578nm_ 0.5 was reached. Filter sterilized solutions of 5-ALA and hemin chloride were added to a final concentration of 500 and 4 µmol L^−1^, respectively. Unless otherwise stated, main cultures were inoculated with 1 mL preculture broth in 100 mL medium in a 500 mL shake flask and vigorously shaken at 200 rpm. Depending on the experiment, induced cells were either cultivated at 23 °C for 19.5 h or at 30 °C for 10 h.

### Outer membrane protein isolation and protease accessibility

Outer membrane protein isolation was conducted as described in literature [69]. To investigate surface exposure of the passenger the protease accessibility test was used. Cells were grown in 100 mL potassium phosphate buffered LB medium at 23 °C for 19.5 h and harvested. Cells from 33 mL culture broth were re-suspended in phosphate buffered saline (PBS). After addition of 50 mAnson U proteinase K the suspension was kept at 37 °C for 1 h. The digest was stopped by addition of 1 mmol L^−1^ PMSF and washing three times with 5 mL of pre-cooled 0.2 mol L^−1^ Tris buffer (pH 8). The cells were then re-suspended in 1.5 mL 0.2 mol L^−1^ Tris buffer and further processed in the outer membrane protein preparation. Samples from the OMPI were boiled 20 min at 95 °C in SDS-PAGE sample buffer with 30 mmol L^−1^ dithiothreitol and separated with a 10 % acrylamide gel. Proteins were stained with Coomassie Brilliant Blue G-250.

### Flow cytometry analysis

To probe surface exposure of the passenger, cells were analyzed by flow cytometry after treatment with fluorescence labelled antibodies. Cells were grown in 20 mL potassium phosphate buffered LB medium in a 100 mL shake flask at 23 °C for 19.5 h. After harvest cells were washed tree times with filter-sterilized PBS. 1 mL of cells (OD_578nm_ 0.2) were treated with 10 µg mL^−1^ monoclonal mouse Anti-myc antibody (ThermoFisher Scientific) for 30 min. Cells were washed three times and then treated with 20 µg mL^−1^ secondary DyLight 488 conjugated goat anti-mouse IgG (H + L) Antibody in the dark for 30 min. After a final round of washing and re-suspension the fluorescence of 50,000 cells was analyzed using a FACSAria flow cytometer (BD Biosciences, USA) at an excitation wavelength of 488 nm.

### Immunofluorescence microscopy

Cells were prepared as described for the flow cytometry analysis. Pretreatment with proteinase K was conducted as described for OMPI. After antibody incubation and subsequent washing steps, cells were re-suspended in 30 µL filter-sterilized PBS, 10 µL applied on a slide and the cover slip sealed with nail polish. Fluorescence images were obtained with a confocal laser scanning microscope TCS SP2 (Leica, Wetzlar, Germany) at 488 nm excitation using an Argon laser.

### Cytochrome c assay

The whole cell assay was performed as described for purified enzymes [35]. Cultivation was performed with 20 mL medium without hemin and 5-ALA at 23 °C in 100 mL shake flask for 19.5 h. Cells were harvested and washed three times in potassium phosphate buffer (pH 7.6.). The assay was conducted in a 96-well microplate with cells at a final OD_578nm_ of 0.25, 50 µmol L^−1^ cytochrome c and 100 µmol L^−1^ NADPH at 30 °C. Absorbance of reduced cytochrome c was measured at 550 nm using an Infinite 200 Pro reader (Tecan Group, Männedorf, Swiss). Concentrations were calculated with the molar extinction coefficient of 21 mmol^−1^ cm^−1^ and a path length of 0.29 cm.

### 7-Ethoxyresorufin-O-deethylation assay

CYP1A2 catalyzes the oxidative O-deethylation of the test substrate 7-ethoxyresorufin to the highly fluorescent resorufin. Cells were harvested and washed three times in 0.1 mol L^−1^ potassium phosphate buffer (pH 7.4). The enzyme reaction was carried out in a 15 mL reaction tube with 0.8 mL total volume containing whole cells (OD_578nm_ 40), 1 µmol L^−1^ 7-ethoxyresorufin and a NADPH regeneration system in 0.1 mol L^−1^ potassium phosphate buffer (pH 7.4). 7-ethoxyresorufin was prepared as 1 mmol L^−1^ stock solution in DMSO and stored at 4 °C protected from light. The regeneration system consisted of 250 µmol L^−1^ NADP+, 1 mmol L^−1^ glucose-6-phosphate and 1 µg mL^−1^ of purified glucose-6-phosphate dehydrogenase from *E. coli* which were pre-incubated at 37 °C before addition. The reaction tube was shaken horizontally at 37 °C for 40 h and protected from light. The assay was terminated by removal of the cells through centrifugation. Depending on the experiment resorufin formation was either determined by HPLC analysis or in a microtiter plate assay.

### Resorufin measurement

For HPLC analysis of resorufin formation a LiChrospher 60 RP-select B column 250-4 (5 µm) from Merck (Darmstadt, Germany) was used in a LaChrom Elite System equipped with a fluorescence detector L-2485. The mobile phase consisted of 50 % methanol and 50 % 0.05 mol L^−1^ potassium phosphate buffer (pH 7). The system was run at a flow rate of 0.6 mL min^−1^. Injection volume was 40 µL. The retention time for resorufin was 7.4 min and for 7-ethoxyresorufin 29 min. Fluorescence was measured at 590 nm using excitation at 550 nm. To save measurement time a sample run was 10 min. Prior to analysis, sample supernatant were diluted with methanol 1:1, centrifuged 10 min at 10,000*g* to remove precipitates and then filtered through a 0.2 µm PTFE syringe filter. Resorufin was quantified with a standard curve ranging from 1 to 200 nmol L^−1.^

For microtiter plate analysis 100 µL of the sample supernatant were analyzed fluorometrically in a 96 well plate using Infinite 200 Pro reader (Tecan Group, Männedorf, Swiss). Fluorescence was determined at 590 nm with excitation at 550 nm. Resorufin was quantified using a standard curve made in the supernatant from co-expression assay samples without substrate treated as described before.

### Phenacetin-O-deethylation measurement

Cells were cultivated in potassium phosphate buffered LB medium additionally supplemented with 1 mmol L^−1^ CaCl_2_. Assay conditions were the same as for the 7-ethoxyresorufin-O-deethylation except that 100 µmol L^−1^ of phenacetin were used. After 40 h incubation 10 µmol L^−1^ of the internal standard 3-acetamidophenol was added and the reaction terminated by addition of 4 mL diethylether to 800 µL L^−1^ of assay volume. The samples were vortexed for 10 min, centrifuged and the water phase frozen. The organic layer was collected, 65 µL 1 mol L^−1^ HCL added to the water phase and the extraction procedure repeated. The collected diethylether was removed under N_2_ stream and samples were dissolved in 150 µL 30 % methanol/70 % 0.1 mol L^−1^ acetate buffer pH 4.

Paracetemol content was determined via the HPLC LaChrom Elite System. 40 µL sample aliquot were separated using a LiChrospher 60 RP-select B column 125-4 (5 µm) from Merck (Darmstadt, Germany) and eluted with methanol/H_2_O (0.1 mol L^−1^ acetic acid pH 4) through a linear gradient from 5 to 30 % methanol for 11 min at a flow rate of 0.6 min mL^−1^. The solvent mixture was then kept constant for 3 min. The column was re-equilibrated for 6 min with 5 % methanol. Absorption was recorded at 250 nm. Retention times for paracetamol and 3-acetoamidophenol were 8 and 10.5 min, respectively. Each substance was quantified with a standard curve ranging from 0.5 to 100 µmol L^−1^. Paracetamol assay concentrations were calculated with regard to the extraction recovery factor determined through the internal standard.

### Luciferin-MultiCYP assay

The luciferin-MultiCYP assay represents a method for the measurement of CYP activity. MultiCYP is a non-selective proluminescent substrate which is convertible to d-luciferin ester by at least 21 CYPs, including CYP1A2 [70]. Protected from light, a 50 mmol L^−1^ MultiCYP stock solution in acetonitrile and a 2 mmol L^−1^ stock solution of d-luciferin in water were made and stored at −20 °C. Cells were grown as described, harvested and washed two times in 0.2 mol L^−1^ potassium phosphate buffer (pH 7.4). Cells (OD_578nm_ 20) were incubated under continuous shaking in 2 mL reaction tubes at 37 °C in 0.1 mol L^−1^ potassium phosphate buffer (pH 7.4) with 50 µmol L^−1^ MultiCYP and 200 µmol L^−1^ NADPH with a total volume of 120 µL. After 24 h, cells were sedimented (2 min, 14000 rpm), the supernatant was collected and incubated at 90 °C for 10 min (to prevent ATP hydrolysis by remaining cell components). Denatured cell components were removed by centrifugation (2 min, 14,000 rpm) and 50 µL supernatant was transferred to white microtiter plates, where 50 µL luciferin detection reagent was added. After 20 min of incubation at 30 °C, luminescence was measured for 10 min and compared to a d-luciferin calibration curve (0–2 µmol L^−1^). Uptake of luciferin by cells was excluded by incubation of d-luciferin with or without cells under assay conditions and subsequent measurement of the resulting luminescence. Heat stability of d-luciferin was determined by incubation of d-luciferin under assay conditions at 90 °C for 10 min.

## Additional files

10.1186/s12934-016-0427-5 Surface localization of CPR and CYP1A2 analyzed by immunofluorescence microscopy.
10.1186/s12934-016-0427-5 Influence of growth conditions on protein expression level.

## Abbreviations

ATP: adenosine triphosphate
5-ALA: 5-aminolevulinic acid
CtxB: cholera toxin B
CYP: cytochrome P450
CYP1A2: cytochrome P450 1A2
CPR: cytochrome P450 reductase
FAD: flavin adenine dinucleotide
FMN: flavin adenine mononucleotide
fXa: factor Xa protease
HPLC: high-performance liquid chromatography
LB: lysogeny broth
NADPH: nicotinamide adenine dinucleotide phosphate
OD: optical density
OMPI: outer membrane protein isolation
OmpT: outer membrane protease T
ORF: open reading frame
ORI: origin of replication
RhaP: rhamnose inducible promoter
TEV: tobacco etch virus

## References

[1] Rendic S, Guengerich FP (2015). Survey of human oxidoreductases and cytochrome P450 enzymes involved in the metabolism of xenobiotic and natural chemicals. Chem Res Toxicol.

[2] Guengerich FP (2008). Cytochrome P450 and chemical toxicology. Chem Res Toxicol.

[3] Hannemann F, Bichet A, Ewen KM, Bernhardt R (2007). Cytochrome P450 systems: biological variations of electron transport chains. Biochim Biophys Acta.

[4] Schroer K, Kittelmann M, Lütz S (2010). Recombinant human cytochrome P450 monooxygenases for drug metabolite synthesis. Biotechnol Bioeng.

[5] Fura A (2006). Role of pharmacologically active metabolites in drug discovery and development. Drug Discov Today.

[6] Urlacher VB, Girhard M (2012). Cytochrome P450 monooxygenases: an update on perspectives for synthetic application. Trends Biotechnol.

[7] Purnapatre K, Khattar SK, Saini KS (2008). Cytochrome P450 s in the development of target-based anticancer drugs. Cancer Lett.

[8] Zelasko S, Palaria A, Das A (2013). Optimizations to achieve high-level expression of cytochrome P450 proteins using *Escherichia coli* expression systems. Protein Expr Purif.

[9] Schüürmann J, Quehl P, Festel G, Jose J (2014). Bacterial whole-cell biocatalysts by surface display of enzymes: toward industrial application. Appl Microbiol Biotechnol.

[10] Gawarzewski I, Smits Sander HJ, Schmitt L, Jose J (2013). Structural comparison of the transport units of type V secretion systems. Biol Chem.

[11] van Ulsen P, Rahman S, Jong WSP, Daleke-Schermerhorn MH, Luirink J (2014). Type V secretion: from biogenesis to biotechnology. Biochim Biophys Acta.

[12] Gawarzewski I, DiMaio F, Winterer E, Tschapek B, Smits SHJ, Jose J, Schmitt L (2014). Crystal structure of the transport unit of the autotransporter adhesin involved in diffuse adherence from *Escherichia coli*. J Struct Biol.

[13] Pavlova O, Peterson JH, Ieva R, Bernstein HD (2013). Mechanistic link between β barrel assembly and the initiation of autotransporter secretion. Proc Natl Acad Sci USA.

[14] Leyton DL, Rossiter AE, Henderson IR (2012). From self sufficiency to dependence: mechanisms and factors important for autotransporter biogenesis. Nat Rev Microbiol.

[15] Detzel C, Maas R, Tubeleviciute A, Jose J (2013). Autodisplay of nitrilase from *Klebsiella pneumoniae* and whole-cell degradation of oxynil herbicides and related compounds. Appl Microbiol Biotechnol.

[16] Kranen E, Detzel C, Weber T, Jose J (2014). Autodisplay for the co-expression of lipase and foldase on the surface of *E. coli*: washing with designer bugs. Microb Cell Fact.

[17] Gratz A, Bollacke A, Stephan S, Nienberg C, Le Borgne M, Götz C, Jose J (2015). Functional display of heterotetrameric human protein kinase CK2 on *Escherichia coli*: a novel tool for drug discovery. Microb Cell Fact.

[18] Salema V, Marín E, Martínez-Arteaga R, Ruano-Gallego D, Fraile S, Margolles Y, Teira X, Gutierrez C, Bodelón G, Fernández LÁ (2013). Selection of single domain antibodies from immune libraries displayed on the surface of *E. coli* cells with two β-Domains of opposite topologies. PLoS One.

[19] Fleetwood F, Andersson KG, Ståhl S, Löfblom J (2014). An engineered autotransporter-based surface expression vector enables efficient display of Affibody molecules on OmpT-negative *E. coli* as well as protease-mediated secretion in OmpT-positive strains. Microb Cell Fact.

[20] Jose J, Betscheider D, Zangen D (2005). Bacterial surface display library screening by target enzyme labeling: identification of new human cathepsin G inhibitors. Anal Biochem.

[21] Benz I, Schmidt MA (1989). Cloning and expression of an adhesin (AIDA-I) involved in diffuse adherence of enteropathogenic *Escherichia coli*. Infect Immun.

[22] Wells TJ, Sherlock O, Rivas L, Mahajan A, Beatson SA, Torpdahl M, Webb RI, Allsopp LP, Gobius KS, Gally DL, Schembri MA (2008). EhaA is a novel autotransporter protein of enterohemorrhagic *Escherichia coli* O157:H7 that contributes to adhesion and biofilm formation. Environ Microbiol.

[23] Sichwart S, Tozakidis IEP, Tesse M, Jose J (2015). Maximized autotransporter mediated expression (MATE) for surface display secretion of recombinant proteins in *Escherichia coli*. Food Technol Biotechnol.

[24] Zhou S-F, Yang L-P, Zhou Z-W, Liu Y-H, Chan E (2009). Insights into the substrate specificity, inhibitors, regulation, and polymorphisms and the clinical impact of human cytochrome P450 1A2. AAPS J.

[25] Laursen T, Jensen K, Møller BL (2011). Conformational changes of the NADPH-dependent cytochrome P450 reductase in the course of electron transfer to cytochromes P450. Biochim Biophys Acta.

[26] Bonina TA, Gilep AA, Estabrook RW, Usanov SA (2005). Engineering of proteolytically stable NADPH-cytochrome P450 reductase. Biochemistry (Mosc).

[27] Schumacher SD, Jose J (2012). Expression of active human P450 3A4 on the cell surface of *Escherichia coli* by autodisplay. J Biotechnol.

[28] Yim S-K, Kim D-H, Jung H-C, Pan J-G, Kang H-S, Ahn T, Yun C-H (2010). Surface display of heme- and diflavin-containing cytochrome P450 BM3 in *Escherichia coli*: a whole cell biocatalyst for oxidation. J Microbiol Biotechnol.

[29] Schumacher SD, Hannemann F, Teese MG, Bernhardt R, Jose J (2012). Autodisplay of functional CYP106A2 in *Escherichia coli*. J Biotechnol.

[30] Yim S-K, Jung H-C, Pan J-G, Kang H-S, Ahn T, Yun C-H (2006). Functional expression of mammalian NADPH-cytochrome P450 oxidoreductase on the cell surface of *Escherichia coli*. Protein Expr Purif.

[31] Yim S-K, Jung H-C, Yun C-H, Pan J-G (2009). Functional expression in *Bacillus subtilis* of mammalian NADPH-cytochrome P450 oxidoreductase and its spore-display. Protein Expr Purif.

[32] Jose J, Bernhardt R, Hannemann F (2001). Functional display of active bovine adrenodoxin on the surface of *E. coli* by chemical incorporation of the [2Fe–2S] cluster. ChemBioChem.

[33] Yoo G, Bong J-H, Park M, Jose J, Kang M-J, Pyun J-C (2015). Electrochemical analysis of autodisplayed adrenodoxin (Adx) on the outer membrane of *E. coli*. Biochim Biophys Acta.

[34] Giacalone MJ, Gentile AM, Lovitt BT, Berkley NL, Gunderson CW, Surber MW (2006). Toxic protein expression in *Escherichia coli* using a rhamnose-based tightly regulated and tunable promoter system. Biotechniques.

[35] Guengerich FP, Martin MV, Sohl CD, Cheng Q (2009). Measurement of cytochrome P450 and NADPH-cytochrome P450 reductase. Nat Protoc.

[36] Yun C-H, Song M, Ahn T, Kim H (1996). Conformational change of cytochrome P450 1A2 induced by sodium chloride. J Biol Chem.

[37] Shen AL, Kasper CB (1995). Role of acidic residues in the interaction of NADPH-cytochrome P450 oxidoreductase with cytochrome P450 and cytochrome c. J Biol Chem.

[38] Kelley RW, Reed JR, Backes WL (2005). Effects of ionic strength on the functional interactions between CYP2B4 and CYP1A2. Biochemistry.

[39] Yamazaki H, Ueng Y-F, Shimada T, Guengerich FP (1995). Roles of divalent metal ions in oxidations catalyzed by recombinant cytochrome P450 3A4 and replacement of NADPH-cytochrome P450 reductase with other flavoproteins, ferredoxin, and oxygen surrogates. Biochemistry.

[40] Kelley RW, Cheng D, Backes WL (2006). Heteromeric complex formation between CYP2E1 and CYP1A2: evidence for the involvement of electrostatic interactions. Biochemistry.

[41] Ahn T, Guengerich FP, Yun C-H (1998). Membrane insertion of cytochrome P450 1A2 promoted by anionic phospholipids. Biochemistry.

[42] Kaur AP, Lansky IB, Wilks A (2009). The role of the cytoplasmic heme-binding protein (PhuS) of *Pseudomonas aeruginosa* in intracellular heme trafficking and iron homeostasis. J Biol Chem.

[43] Anzaldi LL, Skaar EP (2010). Overcoming the heme paradox: heme toxicity and tolerance in bacterial pathogens. Infect Immun.

[44] Létoffé S, Delepelaire P, Wandersman C (2006). The housekeeping dipeptide permease is the *Escherichia coli* heme transporter and functions with two optional peptide binding proteins. Proc Natl Acad Sci USA.

[45] Villarreal DM, Phillips CL, Kelley AM, Villarreal S, Villaloboz A, Hernandez P, Olson JS, Henderson DP (2008). Enhancement of recombinant hemoglobin production in *Escherichia coli* BL21(DE3) containing the *Plesiomonas shigelloides* heme transport system. Appl Environ Microbiol.

[46] Verissimo AF, Daldal F (2014). Cytochrome c biogenesis system I: an intricate process catalyzed by a maturase supercomplex?. Biochim Biophys Acta.

[47] Turlin E, Heuck G, Simões Brandão MI, Szili N, Mellin JR, Lange N, Wandersman C (2014). Protoporphyrin (PPIX) efflux by the MacAB-TolC pump in *Escherichia coli*. MicrobiologyOpen.

[48] Tatsumi R, Wachi M (2008). TolC-dependent exclusion of porphyrins in *Escherichia coli*. J Bacteriol.

[49] Kim D-H, Kim K-H, Isin EM, Guengerich FP, Chae HZ, Ahn T, Yun C-H (2008). Heterologous expression and characterization of wild-type human cytochrome P450 1A2 without conventional N-terminal modification in *Escherichia coli*. Protein Expr Purif.

[50] Vail R, Homann M, Hanna I, Zaks A (2005). Preparative synthesis of drug metabolites using human cytochrome P450s 3A4, 2C9 and 1A2 with NADPH-P450 reductase expressed in Escherichia coli. J Ind Microbiol Biotechnol.

[51] Döhr O, Paine MJI, Friedberg T, Roberts GCK, Wolf CR (2001). Engineering of a functional human NADH-dependent cytochrome P450 system. Proc Natl Acad Sci USA.

[52] Jose J, Meyer TF (2007). The autodisplay story, from discovery to biotechnical and biomedical applications. Microbiol Mol Biol Rev.

[53] Binder U, Matschiner G, Theobald I, Skerra A (2010). High-throughput sorting of an anticalin library via EspP-mediated functional display on the *Escherichia coli* cell surface. J Mol Biol.

[54] Gideon D, Kumari R, Lynn A, Manoj K (2012). What is the functional role of N-terminal transmembrane helices in the metabolism mediated by liver microsomal cytochrome P450 and its reductase?. Cell Biochem Biophys.

[55] Dong M-S, Yamazaki H, Guo Z, Guengerich FP (1996). Recombinant human cytochrome P450 1A2 and an N-terminal-truncated form: construction, purification, aggregation properties, and interactions with flavodoxin, ferredoxin, and NADPH-cytochrome P450 reductase. Arch Biochem Biophys.

[56] Shukla A, Huang W, Depaz IM, Gillam EMJ (2009). Membrane integration of recombinant human P450 forms. Xenobiotica.

[57] Denisov IG, Shih AY, Sligar SG (2012). Structural differences between soluble and membrane bound cytochrome P450s. J Inorg Biochem.

[58] Kim H-J, Lee S-B, Guengerich FP, Park YI, Dong M-S (2007). Effects of N-terminal modification of recombinant human cytochrome P450 1A2 on catalytic activity. Xenobiotica.

[59] Hayashi S, Omata Y, Sakamoto H, Hara T, Noguchi M (2003). Purification and characterization of a soluble form of rat liver NADPH-cytochrome P-450 reductase highly expressed in *Escherichia coli*. Protein Expr Purif.

[60] Jarmander J, Janoschek L, Lundh S, Larsson G, Gustavsson M (2014). Process optimization for increased yield of surface-expressed protein in *Escherichia coli*. Bioprocess Biosyst Eng.

[61] Zgurskaya HI, Krishnamoorthy G, Ntreh A, Lu S (2011). Mechanism and function of the outer membrane channel TolC in multidrug resistance and physiology of Enterobacteria. Front Microbiol.

[62] Langer S, Hashimoto M, Hobl B, Mathes T, Mack M (2013). Flavoproteins are potential targets for the antibiotic roseoflavin in *Escherichia coli*. J Bacteriol.

[63] Abbas CA, Sibirny AA (2011). Genetic control of biosynthesis and transport of riboflavin and flavin nucleotides and construction of robust biotechnological producers. Microbiol Mol Biol Rev.

[64] McAnulty MJ, Wood TK (2014). YeeO from *Escherichia coli* exports flavins. Bioengineered.

[65] Henderson CJ, McLaughlin LA, Scheer N, Stanley LA, Wolf CR (2015). Cytochrome b5 is a major determinant of human cytochrome P450 CYP2D6 and CYP3A4 activity in vivo. Mol Pharmacol.

[66] Berrow NS, Alderton D, Sainsbury S, Nettleship J, Assenberg R, Rahman N, Stuart DI, Owens RJ (2007). A versatile ligation-independent cloning method suitable for high-throughput expression screening applications. Nucleic Acids Res.

[67] Bornscheuer UT, Altenbuchner J, Meyer HH (1998). Directed evolution of an esterase for the stereoselective resolution of a key intermediate in the synthesis of epothilones. Biotechnol Bioeng.

[68] Kovach ME, Elzer PH, Steven Hill D, Robertson GT, Farris MA, Roop Ii RM, Peterson KM (1995). Four new derivatives of the broad-host-range cloning vector pBBR1MCS, carrying different antibiotic-resistance cassettes. Gene.

[69] Park M, Yoo G, Bong J-H, Jose J, Kang M-J, Pyun J-C (2015). Isolation and characterization of the outer membrane of *Escherichia coli* with autodisplayed Z-domains. Biochim Biophys Acta.

[70] Cali JJ, Ma D, Sobol M, Simpson DJ, Frackman S, Good TD, Daily WJ, Liu D (2006). Luminogenic cytochrome P450 assays. Expert Opin Drug Metab Toxicol.

