# Supplementary material for: Co-expression of active human cytochrome P450 1A2 and cytochrome P450 reductase on the cell surface of Escherichia coli
Source: Microb Cell Fact. 2016 Feb 2;15:26. doi: 10.1186/s12934-016-0427-5 (PMC4736170; doi:10.1186/s12934-016-0427-5)
Supplement: Supplementary file 1 — 10.1186/s12934-016-0427-5 Surface localization of CPR and CYP1A2 analyzed by immunofluorescence microscopy. [file 12934_2016_427_MOESM1_ESM.docx]

Figure S1


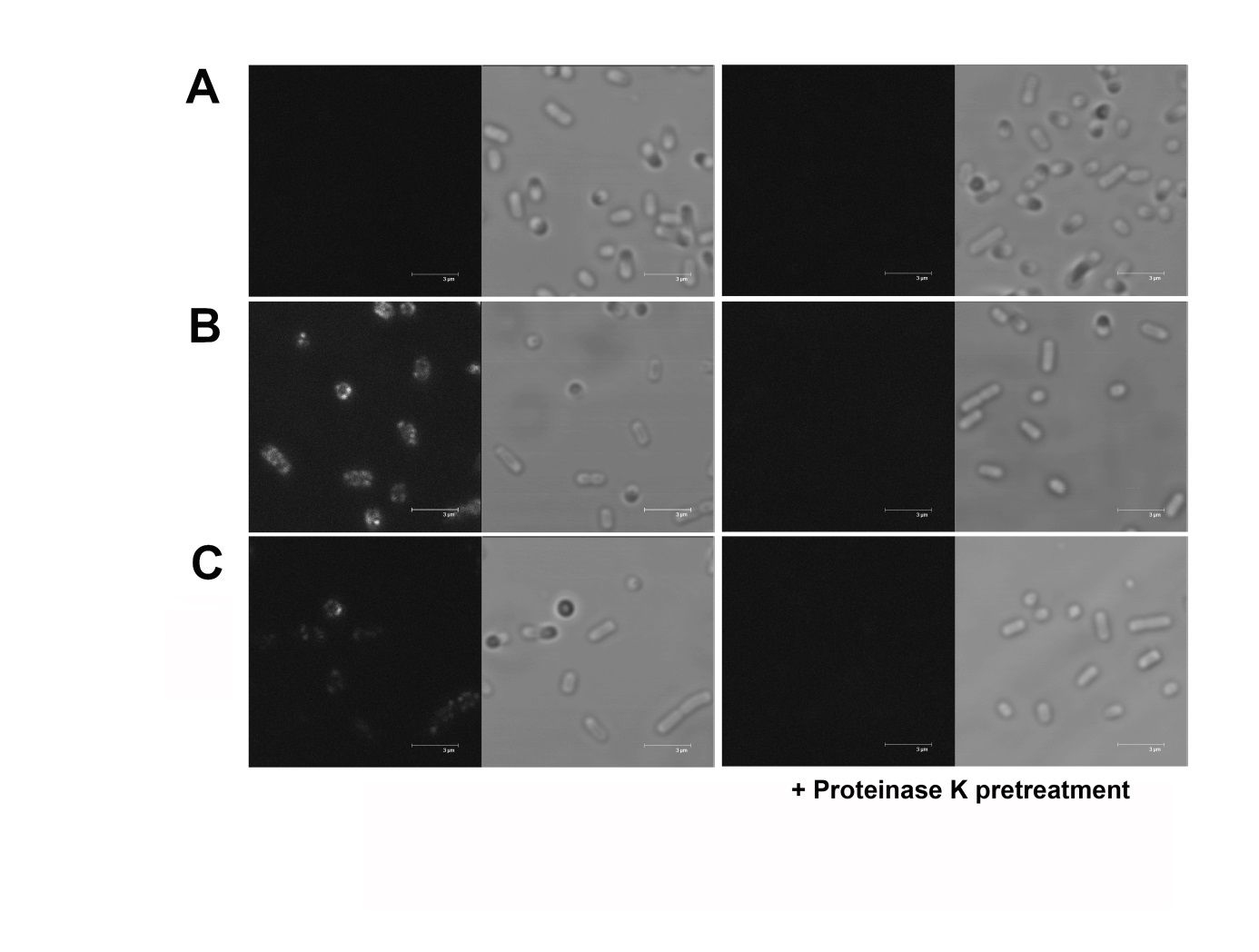


**Surface localization of CPR and CYP1A2 analyzed by immunofluorescence microscopy**

For each cell sample, fluorescence (left panel) and phase contrast graphs (right panel) of the same field are shown. Treatment with a mouse anti-myc antibody and a secondary Dyligh488 conjugated anti-mouse IgG antibody was conducted as described for flow cytometry analysis. A portion of the cells were subjected to proteinase K treatment prior to the antibody labelling procedure as described for outer membrane protein isolation. For microscopy, 10 µL of each cell suspension in PBS were put under a cover slip and sealed with nail polish. A: *E. coli* BL21 (DE3) cells, B: Cells expressing the CPR autotransporter fusion protein with myc-tag (pPQ61); C: cells expressing CYP1A2 autotransporter fusion protein with myc-tag (pPQ62).
