# Supplementary material for: Co-expression of active human cytochrome P450 1A2 and cytochrome P450 reductase on the cell surface of Escherichia coli
Source: Microb Cell Fact. 2016 Feb 2;15:26. doi: 10.1186/s12934-016-0427-5 (PMC4736170; doi:10.1186/s12934-016-0427-5)
Supplement: Supplementary file 2 — 10.1186/s12934-016-0427-5 Influence of growth conditions on protein expression level. [file 12934_2016_427_MOESM2_ESM.docx]

Figure S2


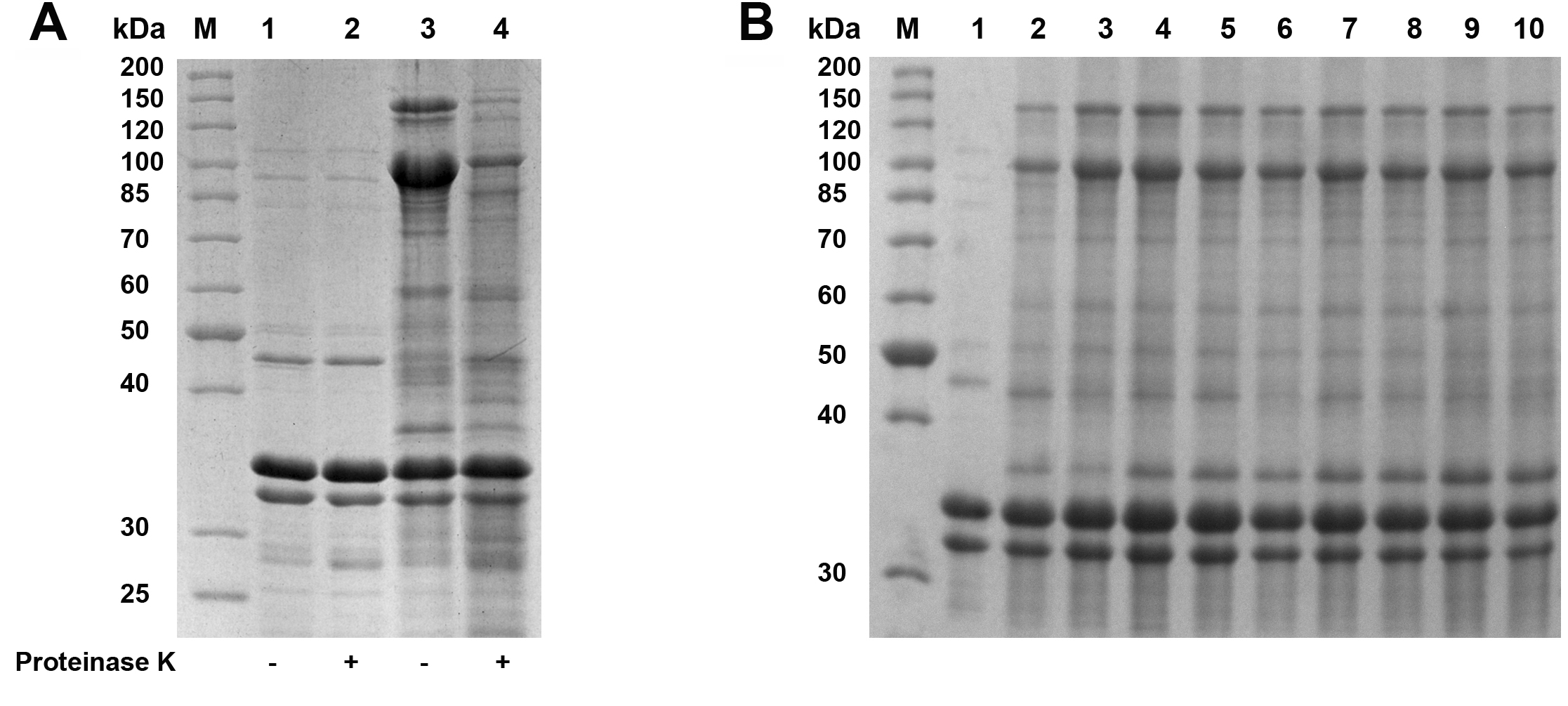


**Influence of growth conditions on protein expression level**

**A:** Protein expression level at 30°C growth temperature assessed by SDS-PAGE of outer membrane protein isolates. Lane 1-2: *E. coli* BL21(DE3) host, Lane 3-4: samples from cells with induced co‑expression of CPR and CYP1A2 fusion proteins. Samples in Lane 2 and 4 were treated with proteinase K prior to outer membrane protein isolation. **B**: SDS-PAGE of outer membrane protein isolations from cells cultivated at different growth conditions. Lane 1: *E. coli* BL21(DE3) host in potassium phosphate buffered LB, Lane 2-10: cells co-expressing CYP1A2 and CPR. Lane 2: in unbuffered LB-medium, 3: in sodium phosphate buffered LB, 4: in mixed sodium/potassium buffered LB, 5: in potassium phosphate buffered LB, 6: with 1 mmol L^-1^ MgCl_2_ in potassium phosphate buffered LB-medium, 7: with 1 mmol L^-1^ CaCl_2_ in potassium phosphate buffered LB-medium, 8: in potassium phosphate buffered LB-Lennox medium with 5 g L^-1^ NaCl, 9: in potassium phosphate buffered LB-Luria medium with 0.5 g L^-1^ NaCl, 10: in potassium phosphate buffered LB-medium without NaCl.
